# Caregiver Reports of Children’s Activity Participation Following Serious Injury

**DOI:** 10.3390/ijerph13070652

**Published:** 2016-07-07

**Authors:** Sandra Braaf, Shanthi Ameratunga, Warwick Teague, Helen Jowett, Belinda Gabbe

**Affiliations:** 1 School of Public Health and Preventive Medicine, Monash University, Melbourne 3004, Australia; belinda.gabbe@monash.edu; 2 Section of Epidemiology and Biostatistics, School of Population Health, University of Auckland, Auckland 1072, New Zealand; s.ameratunga@auckland.ac.nz; 3 Trauma Service, The Royal Children’s Hospital, Melbourne 3004, Australia; warwick.teague@rch.org.au (W.T.); Helen.Jowett@rch.org.au (H.J.); 4 Department of Paediatrics, University of Melbourne, Melbourne 3004, Australia; 5 Surgical Research Group, Murdoch Childrens Research Institute, Melbourne 3004, Australia; 6 The Farr Institute @ CIPHER, Swansea University Medical School, Swansea University, Singleton Park, Swansea SA2 8PP, UK

**Keywords:** trauma, paediatrics, activity restriction, disability, injury, caregivers, qualitative interviews

## Abstract

Paediatric trauma can result in significant levels of on-going disability. The aim of this study was to explore the restrictions on activity participation that children experience following serious injury from the perspective of their caregivers. We performed a thematic analysis of transcripts of semi-structured in-depth interviews with the caregivers of 44 seriously injured children, conducted three-years after the injury, and purposively sampled from a population-based cohort study. Both temporary and on-going restrictions on school, sport, leisure and social activities were identified, some of which were imposed by caregivers, schools, or recommended by health providers. The perceived risk of further injury, physical restrictions, emotional state and fatigue levels were important influences on degrees of activity restriction. Children who were socially less engaged, especially those who were more severely injured, had difficulty making and retaining friends, and exhibited signs of depression or social withdrawal. The activities of pre-school children were strongly regulated by their caregivers, while school age children faced obstacles with participation in aspects such as study, sport, and peer and teacher relationships, affecting learning, school attendance and enjoyment. The findings highlight the need for primary prevention and reducing the impacts of serious injury throughout the continuum of care.

## 1. Introduction

Injury in childhood has the potential to cause devastating short- and long-term consequences. Seriously injured children typically have multiple injuries, require admission to intensive care or urgent surgery. In the years following serious injury, children can experience on-going disability and reduced health-related quality of life, which can restrict their daily activities [1,2]. As participation in activities for children is critical for normal physical and psychosocial development, confidence and identity building, and establishing and maintaining friendships, restricted activities can negatively affect enjoyment, wellbeing, quality of life and childhood development [3,4]. 

Previous studies examining activity restrictions among children with disabilities [4,5,6,7,8,9] have largely focused on children with chronic health conditions such as cerebral palsy, specific activities such as sport, or physical or environmental barriers. Studies of restricted participation in activities for seriously injured children have been limited. A systematic review of eight quantitative studies revealed that participation restrictions on youth with acquired brain injuries (ABI) were associated with greater severity of ABI, the presence of cognitive, sensory, behavioural or motor dysfunction, poor family functioning, and limited accessibility to the environment [10]. However, causes of ABI are aetiologically heterogeneous and include conditions as varied as meningo/encephalitis, brain tumours and strokes. The consequences of ABI following physical trauma can have particular nuances deserving more direct investigation. For children with burn injuries, activity participation was prevented by pain and discomfort, the attitudes of others, concern over appearance, and personal attitudes, fear and concerns [11]. Likewise, children with spinal cord injuries (SCI) and their caregivers reported apprehension to participate in activities owing to fear of physical injury, and concern about experiencing negative social and emotional reactions to their injury and secondary conditions [12].

There is wide variability in the types of injuries children sustain and the potential to impact on activity participation. Research to date has focused on ABI (including traumatic brain injury (TBI)), SCI and burns, but multi-trauma and other types of injury (e.g., orthopaedic, chest and organ injuries) have not been studied. Additionally, most published research on childhood injuries and participation in activities has been conducted using standardised outcome measures and quantitative analysis. Such methods do not capture reported contextual and experiential variations and limit the types of activities recorded. Therefore, the aim of this study was to explore the restrictions on school, social, sport and leisure activities of seriously injured children from the perspective of their caregivers, and the ways in which caregivers managed these restrictions and the ramifications for children’s health and wellbeing.

## 2. Materials and Methods

For this qualitative study, nested within a population-based longitudinal cohort study, 44 semi-structured in-depth interviews were conducted with the caregivers of seriously injured children, three years after the injury event. The study was approved by the Monash University Human Research Ethics Committee (CF14/915-2014000365 approved 25 March 2014) and participating trauma-receiving hospitals.

In the REcovery after Serious Trauma-Outcomes, Resource use and patient Experiences (RESTORE) project, patients are interviewed at three, four and five years following injury using both qualitative and quantitative methods, to gain insights into the long-term impacts of injury. Detailed information about these methods have been previously described [13]. Patients were recruited for the study from the Victorian State Trauma Registry (VSTR), which is a population-based registry that captures data about all major trauma patients in the state. While 138 hospitals provide data to the VSTR, 87% of paediatric major trauma patients are managed at the state’s paediatric major trauma service. Trauma is defined as major if any of the following criteria are met: (i) death related to injury; (ii) an injury severity score (ISS) >12; (iii) admission to an intensive care unit (ICU) for >24 h and requiring mechanical ventilation for at least part of their ICU stay; and (iv) urgent surgery. 

Participants eligible for this study were patients aged 16 years or less when injured, whose date of injury was between 1 July 2011 and 30 June 2012, who survived to hospital discharge and did not opt-off the VSTR. Patients were purposively sampled based on age, gender, compensation status, and metropolitan or regional locations to ensure a range of registry participants were represented. Using this sampling strategy, primary caregivers of paediatric patients were approached to take part in an in-depth interview at the end of the structured three-year follow-up interview. Thus, for this paediatric trauma cohort, interviews were conducted with the patient’s caregiver. As this study involved exploration of in-depth perceptions, only English speaking caregivers were included. Verbal consent was obtained at the start of the interview and all interviews were audio recorded.

Interviews were undertaken between July 2014 and July 2015 by two trained interviewers. The caregivers interviewed were the mother (*n* = 35), father (*n* = 5) or guardian (*n* = 4). Seventy-five caregivers were approached and agreed to an interview, however, in some cases there was difficulty re-contacting the caregiver, the caregiver was too busy or had changed their mind. Requests for interviews ceased after 44 were completed as data saturation was evident. Most interviews ranged from 30 to 60 minutes. A semi-structured interview guide based on the research aims, and previous qualitative research conducted in injured populations, was used to explore caregiver perspectives of their child’s activity participation following serious injury (Table 1). 

Interviews were transcribed and thematically analysed using a framework approach [14]. All interviews were entered into NVivo (version 10, QSR International, Doncaster, UK) to facilitate storage and organisation. The analysis commenced inductively with repeated readings of the transcripts. Themes and issues that arose in the data were noted and grouped based on their content and meaning while retaining their links to the original passages. Using an iterative process, patterns in the data were grouped into emerging themes. A framework of themes and subthemes was subsequently developed and discussed with members of the research team (who had also independently reviewed the transcripts) until agreement was reached. 

## 3. Results

### 3.1. Overview of the Child Patients

All but four of the 44 children were treated at the state’s major trauma service. The median (range) time since injury was 36.0 (35.4–37.7) months. Medicare, Australia’s publically funded universal health care system, was the fund source for hospital care for 68.2% or 30 patients. One quarter were funded by the Transport Accident Commission (TAC), the state’s “no fault” insurer for road injury. The remaining 6.8% were covered by private funds, with one patient of unknown compensable status. The median (IQR) Injury Severity Score was 17 (14–18). Other characteristics of the injured children are shown in Table 2.

The 36-month outcomes from the Pediatric Quality of Life Inventory (PedsQL), a measure of paediatric health-related quality of life, and the King’s Outcome Scale for Childhood Head Injury (KOSCHI), a paediatric measure of primary functional outcomes are presented in Table 2. The PedsQL consists of 23 items designed to measure aspects of physical, mental, social health and role function (school). These items are used to calculate physical health and psychosocial health summary scores. A higher score indicates better health [15]. Caregiver-reported mean scores for both physical and psychosocial health-related quality of life for injured children were slightly lower than the population norm of 86.6 (SD 12.8), and 89.3 (SD 16.4), respectively. The KOSCHI consists of eight categories: death, vegetative state, lower severe disability, upper severe disability, lower moderate disability, upper moderate disability, good recovery, and intact recovery. The 36-month outcomes for the KOSCHI indicate a mix of recovery and disability within this paediatric cohort.

### 3.2. Themes 

Restrictions in activities was a major concern for many seriously injured children with most participants describing on-going concerns three years post-injury. Given important variations in this and related themes by age group, the results are presented sequentially for children aged 3–5, 6–11 and 12–16 years. While the key themes for each age group are specified, detailed descriptions within categories focus on aspects that had particular nuances or differed from other age groups. Quotes to support all themes in each age group are provided in additional excerpts Table S1.

#### 3.2.1. Three to Five Years 

##### Physical Restrictions

Physical limitations existed for nearly all the pre-school injured children, many of which persisted three years after the injury event. Most young children with a TBI also sustained other injuries and experienced cognitive and physical difficulties. The longer-term impacts of problems such as fatigue, impaired balance, strength and coordination on children’s ability to participate in family, leisure and social activities were of particular concern to caregivers, as described in detail by one guardian:

“She’s partially paralysed down the left side of her body. She can’t walk properly. And she does trip quite easily. She can’t run because if she tries to run that’s when she falls. So there’s a lot of issues. She’ll really have problems when it comes to playing any sport activities at school, because she will hurt herself. She’ll want to do it, she’ll want to join in but she won’t have the ability to do it.” (*3–5 years, female, multiple injuries; Guardian*).

##### Postive Attitudes 

Notwithstanding significant physical restrictions, nearly all pre-school injured children were eager to participate in activities. Caregivers frequently commented on the children’s positive attitude and determination to work around limitations. Caregiver attitudes, and the attitudes of other young children also played an important role in the injured child’s participation. Some caregivers wanted to always be present when their child’s was involved in activities, while others took a less regulated approach. For example, one caregiver described how despite a limb amputation their young child had a strong will, and the freedom, to try different things: 

“If his brother’s doing it, he’ll do it... But then other times he just likes the challenge. He’ll give it a go… but he’s not a kid that will stand back and say I can’t do that. He’ll participate to the best of his capability... we still put him in to things. So (child’s name) plays tennis, but he just plays with one hand. But he loves it. We don’t want to hold him back from anything.” (*3–5 years, male, multiple upper extremity injuries; Mother*).

The same mother continued to describe how other young children routinely accepted her son’s disability to enable participation:

“All these kids are standing around him, and I sort of went over and they were just asking what happened. They were just little boys and I said, ‘What’s going on?’ And they said, ‘What happened; is it serious?’ (Child’s name) had already said, ‘I’ve had an accident.’ Two-second wonder and then they’ve turned around and said, ‘Oh, well, it’s good that he can still play football’, and off they went.” (*3–5 years, male, multiple upper extremity injuries; Mother*).

Other caregivers described how children with severe disabilities found ways of engaging in recreational activities even when they were physically incapable of directly participating in more rigorous, pursuits such as sport. 

“(Name of injured child’s sibling) does athletics and Auskick and I’d like her to do some of that sort of stuff but she can’t. More often than not she comes. She doesn’t participate, obviously, but she’ll just come and she’ll just stay with me. And she enjoys it. Because it’s like a little outing for her, too.” (*3–5 years, female, multiple injuries; Father*). 

##### Missing Out

Injured preschool age children spent little time without their caregivers. Not yet at school, and many still recovering from their injuries, caregivers were very involved in regulating their child’s activities. This was sometimes frustrating for young children who were keen to take more active roles than they were allowed by caregivers. One guardian described her struggles in enforcing longer-term activity restrictions advised by clinicians to protect the child from further injury while contending with a pre-school child who perceived these restrictions as unfair, being unable to understand the reasons. 

“She’ll never be allowed to go on a jumping castle or a trampoline for the rest of her life. So that affects some of the play things that she could get enjoyment out of. We had a party on Saturday and there was a jumping castle and she wanted to go in it. And you have to say no. So she just looks at you and drops her lip and starts to cry… Which is quite disappointing for her that she can’t join in the activities that the other kids are playing.” (*3–5 years, female, multiple injuries; Guardian)*. 

While on-going disability restricted the activities of many pre-school children, a small number of children with cognitive changes post-injury also noticed they missed out on activities due to social exclusion from play.

“A couple of weeks ago now she was saying she had to play with two little girls… yet she came home all upset because they said that she wasn’t allowed to play with them anymore and that they didn’t want to be her friend… I think it was because she was having trouble following the rules of their game. I said that’s okay, so what did you do? … So we’ve tried to make her resilient.” (*3–5 years, female, traumatic brain injury; Mother*).

#### 3.2.2. Six to Eleven Years 

Injured children aged six to 11 years old exhibited some similarities with their younger counterparts with activity restrictions due to on-going disability and limitations imposed by caregivers. However, this was not routinely perceived by this age group as “missing out”, given most older children understood the reasons for activity restriction. While there were notable exceptions, many injured children also demonstrated a positive attitude towards participation. A major environmental influence for children of this age group was their presence at school.

##### Physical Restrictions 

Similar to injured pre-school children, physical limitations existed for nearly all school age injured children. This older age group, however, more frequently reported enduring issues with fatigue, headaches and joint pain, limiting school, sports, leisure, and social activities.

“He’s got really sore knees and he’s always complaining of a sore hip. He hasn’t played sport since he’s been in the accident.” *(6–11 years, male, multiple injuries; Mother)*. 

##### Negotiating Activities at School

Immersion in the school environment placed different demands on injured children as they were expected to participate in learning and sport, and develop peer and teacher relationships away from their caregivers. Accordingly, the consequences of injury now affected school participation in aspects such as learning, attendance and enjoyment.

Some caregivers reported that while their child was keen to attend school, a loss of confidence in aspects of school activities occurred when they feared re-injury. The period of activity withdrawal varied, but was enduring for some.

“…so it has affected her physical activity for a very long time, I think over a year, she was very paranoid…. I would get so many phone calls from the school saying, (child’s name) in the sick bay; she’s scared she’s hurt her liver again; she’s crying; she’s upset.” (*6–11 years, female, abdominal injuries; Mother*).

Given the growing importance of peer relationships in this age group, some injured children experienced restricted social activities related to physical dysfunction, social withdrawal, or loss of confidence. Others were socially excluded from participating with their peers due to behaviours seen as problematic, especially following head injury. The types of behaviours of particular concern included: standing too close or needing to always be near others, saying inappropriate things, acting impulsively and with anger, and hitting others. The mother of a child with a TBI described the consequences of her child’s injury on social networks and how this interacted with her learning activities.

“The social and behavioural problems are a big impact on the other children and how they have to cope and deal with what (child’s name) throws out their way… it’s just a never-ending cycle of ‘I’ve got a new friend and I’ve had a fight with this friend.’… sometimes it’s questionable whether she even has somebody to go to when she gets to school, because of what happened the previous day or the previous week… If something happens in the playground it always affects what happens in the school classroom.” (*6–11 years, female, traumatic brain injury; Mother)*.

The mother cited above, further explained the cognitive and physical demands of school on her child and the subsequent development of headaches and fatigue. Fatigue associated with school attendance was common among all injured children, but reported by caregivers of all children with a TBI. Many caregivers reported attempting to regulate activities to lessen secondary problems.

“After her school day, and to continue on to do a sport as well, is a hard thing for her to cope with all in one. Her push and her drive to do those kind of things is fantastic but it causes headaches afterwards, which causes more frustration on top. So we’ve held off on putting her into sport… after school. She’s capable and able doing sport, and running, etcetera. But the fatigue side is the biggest thing.” (*6–11 years, female, traumatic brain injury; Mother*).

##### Caregiver Involvement in Their Child’s Activities at School

Caregivers’ approach to supporting children in this age group sometimes took the added dimension of direct involvement in advocating for issues that optimised their child’s engagement in learning activities. This is apparent in the comments from a mother of a child with a TBI who took a particularly proactive approach:

“…different things help her at school as well and having to communicate with teachers’ aides and explain to them what (child’s name) experiencing and how fatigue works and how the actual injury works and what the injury is, and to clarify that there is because there are not many physical symptoms that she shows, they’re all cognitive and social aspects…That’s what the neuropsychologist was there for in the first place, was to help me explain to them what was going on and what to expect and what could happen and how it happened and what it is and why it’s happened.” (*6-11 years, female, traumatic brain injury; Mother*).

Caregivers of children without TBI similarly discussed the need to advocate for interventions such as integration aides, access for rehabilitation specialists, and reduced school sports involvement. A mother of a child with a burn injury recalled advocating for tolerance and understanding from the child’s peers and teacher.

“I sent them photos, to his teacher, before he went back to school so the kids knew what to expect. And I went in and sat down with the teacher in front of the class, because the kids didn’t even know about it, and explained to them about his injuries and what he was suffering at the time.” (*6–11 years, male, burn injuries; Mother)*.

However, caregiver efforts to change the school environment for optimal participation in learning activities for their child were not always successful.

“It’s getting teachers to understand, sort of thing. But they don’t have to treat it (child’s behaviour) like he’s a naughty kid. Really, he just needs to stand in the room for a minute and he’s right. So it’s just a school policy. That’s the way it is, they don’t want kids wandering around so they keep them in the classroom now, so it hasn’t worked in (child’s name) favour at all.” (*6–11 years, male, multiple injuries; Father*).

While most caregivers expressed considerable concern about the apparent unwillingness of schools to accommodate their child’s particular needs, a few reported notable successes in the process of transitioning back to school post-injury.

“They supported her very well and helped her with her reintroduction into the school after the accident and things like that. They were very supportive and made sure they behaved in a manner that didn’t highlight the fact that she had actually been involved in an accident.” (*6–11 years, female, multiple injuries; Father*).

##### Passivity and Disconnections from Activity and Social Participation

While most school age injured children had a positive attitude towards school attendance and engagement, caregivers reported that a small number of children were disconnecting from school activities with wide-ranging implications for many spheres of life.

“Mainly just his interaction with staff, the teachers, the students, is quite poor. And his academic level seems to be dropping compared to when he first came… report card shows a decline in his academic progress. I’m not sure how well he focuses. I think it’s affecting his role as a student in the classroom and his interactions in the classroom… and this term they put him on a special program to see if they can get him to focus on school and stop mucking around so much in class.” (*6–11 years, male,* *traumatic brain injury; Guardian*).

The same guardian described similar withdrawal behaviours extending into home life as the injured child pursued secluded and sedentary activities.

“… to participate in anything physical... lots of encouragement to do that. Otherwise it’s just the Xbox; it has really become his life now. He has Xbox live, that means he has these cyber friends… He can be on it all night at times. I’ve got to go in sometimes two, three in the morning to tell him to get off his game, go to bed. So that has impacted on his life greatly.” (*6–11 years, male,* *traumatic brain injury; Guardian*).

Extended time away from school to recover from injuries caused a small number of children to become indifferent to learning activities. Sometimes these behaviours affected the entire classroom learning environment.

“I just think he’s lost interest in school; he can’t do the work…. It’s just because of the injury and he got behind… he knows how to do the work, he just can’t do it. So when he can’t do it he distracts the other kids” (*6–11 years, male, abdominal injuries; Mother*).

Caregivers also reported a number of injured children substituted their sporting activities with non-contact sports or hobbies such as learning a musical instrument or computer games. While many caregivers welcomed these changes, as they were perceived to be low risk, some were concerned about the passive nature of their child’s newly adopted activity.

#### 3.2.3. Twelve to Sixteen Years 

Notwithstanding many similarities to the six to 11 year age group, a striking aspect of this older age group of injured children was their increasing independence, and in most cases, growing separation from their caregivers.

##### Varying Effects on Adolescents’ Independence and Caregivers’ Sense of “Powerlessness”

In general, caregiver reports suggest that despite their injury, the attitudes and behaviours of most adolescents were suggestive of increasing independence from their caregivers. However, there were notable exceptions. One mother explained how her daughter’s depression since the injury had increased her dependence on family and her presence at home. 

“She doesn’t leave my side. She has very frequent afternoon naps. She’s always tired. No social life. Since she left school she doesn’t mix with anybody… she has a lot of depression.” (*12–16 years, female, multiple injuries; Mother*).

More commonly, caregivers reported a high risk of conflict if they exercised too much caution or disagreed with their child’s decisions. One mother recounted her discontent at not being able to convince her son to access support from health providers. 

“You can’t go through something like that and not have some repercussions I don’t think, but he won’t tell you about it, as in flatly refuse to go and see a psychologist or ... as soon as you mention something along those lines he just shuts up and nope.” *(12–16 years, male, multiple injuries; Mother)*.

A key theme expressed by caregivers about injured children in this age group was feeling powerless to influence their decisions. One mother discussed her sense of resignation at being unable to govern her injured son’s activities.

“We are different people now. Before, I was never ever scared of anything. I wanted him to do anything he wants to do, but now I’m more cautious. We let him do what he wants, but we always tell him what can happen. … So now we tell him and then he always tells us that we spoil things for him. But, anyway, the other day he did skydiving.” *(12–16 years, male, head and facial injuries; Mother).*

Caregivers’ also expressed their powerlessness in helping their child to overcome significant challenges in unfriendly social environment. They found themselves to be witnesses to how insensitive actions by peers had damaging emotional effects on their adolescent children, some of whom reacted with rage, became deeply withdrawn, spent increasing amounts of time isolated or with family, and no longer wanted to attend school. Hostile social environments were experienced by adolescents with or without a TBI.

“When he went back to school, things didn’t work out … his friends had deserted him… He was bullied... he was spat upon.” (*12–16 years, male, traumatic brain injury; Mother)*.

This mother, like many of the other caregivers, attempted to mitigate the negative effects of social activity restriction by implementing strategies to facilitate activity participation. These strategies, however, differed in success.

“Young men, they don’t understand after someone’s had an injury don’t know how to cope with it. And after he came home I invited them all over, just for them to at least get reacquainted, but it just fell through…. That was the worst. That really, really was. Because, I mean, we all knew ... apart from our immediate family, we needed other outsiders to be involved with our lives as well, but he hasn’t got that at all…. I’m trying to engage him, like with these other social groups; like a chess club, or photography course, but he just puts up the brick walls.” (*12–16 years, male, traumatic brain injury; Mother*).

##### Concerns for the Future

Several caregivers of severely injured adolescents expressed concern about their child’s future activities and career paths. However, others commented on remarkable degrees of determination and resilience on the part of their children, as indicated by the following comment about how one adolescent identified a career pathway directly and positively influenced by her numerous activity restrictions.

“She does have an aide at school… It’s not only her inability to process information, she was also right-handed before the accident, and the injury is to the left side of her brain, so she lost the whole right side of her body, so she’s had to learn to rewrite with her left hand, which is very taxing for her…She wants to work with disabled children, or disabled people, and so we were able to get her a placement … which is just brilliant for her, because it’s given her a reason to do things, and the reason for her to go to school, is also to get the certificate that she needs to be able to go on with the work that she wants to do.” (*12–16 years, female, head and other injuries; Mother*).

## 4. Discussion

In this large qualitative study based on caregiver perceptions, nearly all children experienced some restriction on their activities following serious injury. Consistent with previous literature on children with disabilities, ABI, SCI or burn injuries [11,12,16,17], many seriously injured children were confined in their activities inside and outside the home. While a number of restrictions were temporary, many were enduring, and frequently originated in combination from multiple sources. Limited engagement in activities reduced quality of life and resulted in lost opportunities to build social networks, improve physical function, develop independence, and experience enjoyment [4,16,18]. Importantly, this study also revealed how children and their caregivers drew upon multiple sources of resilience and resourcefulness with a view to overcoming obstacles in the physical and social environment. Hence, it is clear from our findings that accessible interventions with a goal of preventing secondary injury are important for children who survive major trauma.

The intersection of childhood development and recovery from serious injury was highlighted in our study. The themes of activity restriction from physical, cognitive and environmental limitations traversed all age groups, however, the social consequences and environmental contexts differed. Activity restrictions in many pre-school children incited a sense of social injustice, and caused negative emotional reactions and a reduction in the enjoyment of activities. This highlights the importance of young injured children feeling empowered to engage in alternative recreational opportunities. Further, as pre-school children are typically still under the watchful eye of their caregivers, central to participation for these young injured children was the attitude of their caregivers. Our findings emphasize the importance of caregivers providing and enabling opportunities for injured pre-school children to independently resolve issues and work around their injury limitations while in a safe environment.

Participation in school and learning activities are an important component of developmental and academic success [19,20]. Activities at school involved new challenges for injured children over six years of age, as they were expected to navigate sport activities, peer and teacher relationships, and set learning outcomes in an environment away from their caregivers. While most injured school age children possessed a positive attitude towards school activities, the impact of the injury affected the academic performance of many. These results are comparable to children with special health needs and moderate to severe TBI [21,22]. Further, the physical, psychological, social, behavioural and environmental issues that arose at school highlights the impact of serious injury on learning, even in the absence of learning disability. Many caregivers actively advocated modifying their child’s school environment. These attempts to improve participation in school activities for their child were sometimes met with indifference by schoolteachers. This finding has direct implications for improved collaboration between children, caregivers and schools to maximise the social and academic engagement of injured children. This issue is of particular importance for connectedness to school, which is a protective factor for child and adolescent health, education, and social wellbeing [23,24].

In contract to younger school age children, passivity and disconnections from activity and social participation manifested clearly in the adolescent age group as depressive symptoms and acute social withdrawal. Adolescents who were bullied or experienced depressive symptoms were more likely to disengage from school. Children without a sense of connectedness to their school environment can have detrimental emotional and mental health outcomes [24]. Previous studies have found that negative attitudes from others (real or perceived) are associated with reduced social functioning, activity involvement and quality of life in children with disabilities [6,11,25,26]. Further, post-injury depression has also been identified as a significant and frequent complication for adolescents involved in major trauma events [27] that can result in social withdrawal [28]. Increasing independence of adolescents, however, often left caregivers feeling powerless to intervene, to either support their child with professional psychological assistance, or to keep them attending school. Collectively these findings have implications for early psychological assessment, timely intervention to support positive mental health and foster school connectedness, and for health professionals and schools to share the burden with caregivers in actively addressing the issues faced by school age injured children. 

Activity restrictions profoundly affected the day-to-day social opportunities and activities in injured children. Considered an essential part of a child’s development, a lack of social participation in childhood can adversely affect academic progress, social growth and psychological outcomes in the short and long term [4]. Similar to studies examining social impairment and TBI, barriers to social activity participation in seriously injured children with TBI predominately stemmed from challenging behaviours [25] that led to social exclusion. Conversely, injured children without a TBI experienced social activity restrictions primarily as a result of reluctance to participate in activities owing to physical dysfunction, depressive symptoms, loss of confidence, or fear of re-injury. These differing reasons for social activity restrictions on seriously injured children have implications for preventive education and interventions designed to support participation in activities following injury.

We identified important sources of resilience across all age groups influencing children’s activity participation post-injury. Some children positively reshaped their approach to activities and met the challenges of their injury restrictions with a sense of self-efficacy, determination, persistence and problem-based coping strategies. Participation in activities was also supported by family and community networks, a finding reinforced by others [29,30]. Reflecting individual differences in ways of coping, the recovery trajectory for activity participation was lengthy for some children, while it involved only sedentary, isolating or passive activities for others. Resilience as a process of adjustment has also been noted in seriously injured adults [31]. The implications of restrictions on physical activity, irrespective of its origins, has repercussions for the development of secondary conditions (such as social isolation, weight gain, inactivity, or depression) [32], and underscores the need for targeted and accessible interventions to promote resilience, health and wellbeing.

As the pathway to activity participation for injured children can be complex, important implications for the development of timely and effective interventions arise. Our results indicate that a collaborative approach encompassing the injured child, the caregiver and the child’s social and physical environments is necessary to mitigate the negative consequences of activity restrictions. This resonates with the principles of a social model of disability which positions all people (with and without a disability) as collectively accountable for reducing disabling barriers [33]. Alongside attention to necessary adaptations of environmental structures and processes (e.g., at school), support for caregivers could be facilitated by health professionals through the provision of information about current and expected stages of development for an injured child. For example, for pre-school children information could be provided about how to balance their freedom to explore injury related problems while also maintaining a safe environment. While there was evidence that some caregivers were engaging with health care professionals for advice about children with on-going issues, many were not. Long-term interaction with health care professionals may be required to support caregiver decision-making and to maintain current knowledge of available community programs and resources that foster enjoyment and expand peer networks for injured children.

To manage the different types and consequences of serious injury, schools and teachers require specific knowledge and skills. Sufficient training tools and appropriate supports are necessary to prevent the exclusion of an injured child’s needs. A collaborative model of coordinated care to optimise school activity participation post-injury could involve a health professional liaison that is engaged with a child’s learning and caregiver while in hospital or rehabilitation, who then continues this work as the child transitions back to school. This would enable the liaison to act as a resource for the child’s teacher, thereby reducing the risk of an injured child’s needs being marginalized. Such a role would reduce the burden on the many caregivers who currently perform this function, and would also ensure equity for all injured children. Caregivers from different linguistic and cultural backgrounds to those interviewed in this study, or those of low socioeconomic status or poor education, might be less likely to advocate for their child’s special needs. Many successful programs also exist that have improved school reintegration for children with TBI [34]. These interventions could also be adapted for use in children with other injuries.

School programs aimed to address teasing and bullying at schools are vital for changing attitudes towards children with disability. Program goals should be multi-focused, address teacher, caregiver and injured children with the aim of educating all children about primary injury prevention and injury consequences, as well as building coping skills and resilience in injured children. Such programs are necessary to ensure an injured child’s self-confidence and self-concept are not eroded, and that psychological problems are prevented or mitigated [26].

### Limitations

While this study offers important insights into restrictions on the activities of seriously injured children, when interpreting the findings of our study, the following limitations need to be considered. This study only involved those with serious injury (most of whom were treated in the state’s major paediatric trauma facility), and therefore the findings may not be applicable to less severely injured children or those treated at a non-major trauma service. As the study involved exploring caregiver perspectives over a three-year period, it is possible that caregivers’ memories have faded. However, caregivers often identified when they felt their memories were incomplete. This study only included the perspective of caregivers, which may differ from the perspectives of injured children. The views expressed by caregivers in this study could represent those with a particular interest in problems and challenges faced following serious injury. Limited information was collected about the caregivers aside from their relationship to the injured child and their English language proficiency. Future research should address these limitations including the voices of children and drawing on experiences of children and families from non-English speaking backgrounds (who can face many challenges negotiating health and education systems [35]).

## 5. Conclusions

This qualitative study based on caregiver perceptions revealed that many children experienced a reduction in the number and frequency of activities, as well as a change in type and level of activity engagement, even three years after the injury event. These were associated with complex, interlinked and sometimes long-lasting physical, social, emotional, cognitive and behavioural consequences. These were often recognised as key drivers influencing restrictions on injured children’s activity engagement. These findings highlight the importance of primary prevention of injuries and preventing secondary harms throughout the continuum of care. There is an obvious need for early and targeted interventions to reduce the impacts of injury and improve health and wellbeing in seriously injured children.

## Figures and Tables

**Table 1:** Interview guide.

Interview Questions
What impact has your child’s injury had on home life?
Has the injury affected their ability to do things at home in any way?
How has it impacted on the rest of the household?
What impact has the injury had on your child’s participation in activities outside your home life?
What factors influence your child’s participation in activities?
Has the injury affected your child’s relationships with family members (e.g., parents, siblings), other relatives or friends or in any way?
What impact has their injury had on them emotionally?Has the injury affected their schooling?
What impact has the injury had on your child’s general health?
Have they had any other problems as a result of their injury?
Has your child experienced any difficulties in their environment with the attitudes of others or with policies or services?

**Table 2:** Characteristics of child patients.

Descriptor	Group	Mean (SD)/n (%)
Age		Mean (SD)
		8.8 (5.3)
		*n* (%)
	3–5 years	16 (36.3)
	6–11 years	12 (27.4)
	12–16 years	16 (36.3)
Gender		*n* (%)
	Male	26 (59.1)
	Female	18 (40.9)
Mechanism of injury		*n* (%)
	Fall	10 (22.7)
	Motorcycle	6 (13.6)
	Struck by/collision with object or person	6 (13.6)
	Motor vehicle	5 (11.4)
	Other causes including pedal cyclist, pedestrian, horse-related, fire, flames, smoke	17 (38.6)
Discharge destination		*n* (%)
	Home	35 (79.6)
	Inpatient rehabilitation and other	9 (20.4)
36 month outcomes	KOSCHI *^,#^	*n* (%)
	Intact recovery	17 (38.6)
	Good recovery	8 (18.2)
	Upper moderate disability	6 (13.6)
	Lower moderate and upper severe disability	13 (29.6)
	PedsQL ^^^	Mean (SD)
	Physical health summary score (*n* = 43)	83 (21.0)
	Psychosocial health summary scores (*n* = 35)	82 (19.3)

* Data has been collapsed from eight outcome categories; ^#^ The King’s Outcome Scale for Childhood Head Injury; ^^^ Pediatric Quality of Life Inventory.

## Supplementary Materials

The following are available online at www.mdpi.com/1660-4601/13/7/652/s1, Table S1: Additional excerpts.
