# Supplementary material for: Caregiver Reports of Children’s Activity Participation Following Serious Injury
Source: Int J Environ Res Public Health. 2016 Jul 7;13(7):652. doi: 10.3390/ijerph13070652 (PMC4962193; doi:10.3390/ijerph13070652)
Supplement: Supplementary file 1 [file ijerph-13-00652-s001.pdf]

Supplementary Materials: Caregiver Reports of Children's Activity Participation Following Serious Injury

Sandra Braaf, Shanthi Ameratunga, Warwick Teague, Helen Jowett and Belinda Gabbe

Table S1. Additional excerpts.

3 to 5 Years
"Our kids were playing basketball…she's such a little thing, I was probably more worried she'd just get hit by the basketball; the basketball is almost bigger than her. 'I want to play; I want to play.' She probably wasn't ready to play the game but she'd still come down and train with the kids, like she really enjoys that and participating, and she went really well." (3–5 years, female, head and other injuries; Guardian).
"I am probably a lot warier of allowing her to be in the care of family without me. They used to go and stay at my mum's a fair bit, they haven't been to stay at my mum's since the accident. Just not confident that mum will follow her routines. She copes best when there is a very strict routine…And I suppose I put all that stuff into the reassessment of our life; that extra fluffy bits were nice, but we don't have time for them anymore. We've become very focused on what goes on in our four walls. I don't push myself to go to all the birthday dinners and family get-togethers and stuff because it sometimes just doesn't suit our routine." (3–5 years, female, traumatic brain injury; Mother).
"She looks relatively normal, apart from having glasses, and she occasionally gets the blank expression on her face because she has a bit of trouble keeping up with the conversations around her. When there's too much input she just gets confused. I can see that in her and I can pick when it's happening and I can intervene, but when she's out in the big wide world she can just get lost type of thing." (3–5 years, female, head injuries; Mother).
"He couldn't get back on the motorbike, he couldn't do anything, all because of his liver; so he had to take it really easy. He wasn't allowed to get in the tractors, because he loves his tractors, and he couldn't do any of that. So he used to go down to the tractors to just stand there and look at them." (6–11 years, male, abdominal injuries; Mother).
"And playing on playgrounds, she can but she has to have someone behind her with her always. She can't just get up and play. Before the accident she did dancing for two years… she can't do that anymore. She can't joint a sports team and play sport. I think that impacts, like her being able to join in a group and play, and physically..., she can't go to a school program, a school holiday program where she's just left, so that impacts her ability to socialise with kids." (3–5 years, female, multiple injuries; Mother).
"It hasn't affected her socially except for the time she missed being away from children. So she was away from school for about a year. So I think that has delayed her because she was with adults that whole time. So I do see things that other kids do better than her that she will eventually get on to in making friends and that sort of thing." (3–5 years, female, burn injury; Mother).
"Socially she tends to get on with children that are ... she's seven, probably some of the children she gets on with better are like three and four. And I think that's because she can navigate the games and the rules she plays without really having to process it." (3–5 years, female, multiple injuries; Mother).

Table S1. *Cont.*

6 to 11 Years
"Her learning capabilities are nowhere near what she was, and she's very far behind at school. She has a full-time teacher's aide to help support her but we found she needs more support in her cognitive and social aspects, and she does ... But her schooling, her education, she's very far behind." (6–11 years, female, traumatic brain injury; Mother).
"He can't run; he tried to run, but he is mainly on the computer. He played a little bit of footy and that beforehand but he sort of ... his mates and that are in the computer and going for walks and that anyway." (6–11 years, male, multiple injuries; Father).
"He doesn't do things because he's scared that he might hit his head or hurt himself." (6–11 years, male, multiple injuries; Mother).
"He won't participate. If he does he just goes the once. I had a talk him about maybe in participating in soccer for a term, and paid for his term fees, and he only went the once and the next week he just said I don't want to go." (6–11 years, male, head injuries; Guardian).
"I just think that we had a lot of issues where we went back to school where they were making him go outside and play with the kids when, clearly, he's just been out of hospital. This was after going to the school and explaining to them what had happened. They knew, but just to the extent of he's not allowed to play basketball because he'd obviously suffered head injuries and we just need to be very careful. I felt a little bit that they excluded him without realising the fact that he was sick…. They just didn't accommodate him for his situation." (6–11 years, male, multiple injuries; Mother).
"I would never put him on a public transport train or bus by himself without someone who knows him there to back him up, because people can be cruel." (6–11 years, male, burn injuries; Mother).
"(Child's name) a little sporty kid. So he's sort of got a leg injury, which has caused him stopping the growth in one leg. So as much as I try and think maybe he should do something else he's playing cricket this year… he could barely run between the wickets a year ago, but he still insists on playing." (6–11 years, male, multiple injuries; Father).
"I worry about him when it's summer because he has to stay outside at school, and then it triggers him to get headaches, and then he'll have to stay home the next day if he had a headache the whole day… He gets headaches a lot more when it's hot." (6–11 years, male, multiple injuries; Mother).
**12 to 16 Years**
"It doesn't happen very often (social activities). It'll depend on how she's feeling, what mood's she's in, how she's coping with situations, who will be there when she goes there, and how they'll ... she might be meeting a friend, but who will that friend be with, will have an impact on her." (12–16 years, female, head injuries; Mother).
"He was pretty un-keen about skiing for a while. The first year after the injury we actually went to Europe and made him ski…. and having been a very good skier he was very scared and didn't want to." (12–16 years, male, abdominal injuries; Mother).
"He never returned to sport because I wouldn't let him play football over the winter, because it happened in the summer and then when it came to football season I just said no, and he didn't really want to, he didn't want to play anyway." (12–16 years, male, head injuries; Mother).

Table S1. *Cont.*

**12 to 16 Years**
"Both mum and dad are very, very protective of him now. More so than what they were previously… I've kept him from doing a few things. He would have been ... if I didn't say no he would have gone to a concert about a week or so later. It was like, no, you're not. So I sort of stopped him for about two months, but that was about as much as I could do. I didn't have an argument because there was really nothing stopping him from doing it." (12–16 years, male, multiple injuries; Mother).
"I think maybe the schools still need to have more education, just because you don't look different, doesn't mean you haven't got an underlying medical condition that you can't see, that does impact your schooling." (12–16 years, male, multiple injuries; Father).
"Everybody knows in this town what happened to her, and yet there are still kids, and even down to one of the girls that was on her (sport) team the day that this accident happened, who picked on her because she's got a limp. And that just completely ... there's no part of my brain that can accept that." (12–16 years, female, head injuries; Mother).
"With the footy and things like that, he'd only play like half a game… Maybe play the second and fourth quarter or something like that. Just so he got the rest and everything that he needed, because he would just get puffed out pretty quickly." (12–16 years, male, burn injuries; Father).
"One who has been very good is the speech pathologist, who's been amazing. She's organised education sessions with the teachers at (child's name) school. I speak to her on the phone. She's been very adaptable with what we've been doing." (12–16 years, male, head and other injuries; Mother).

© 2016 by the authors; licensee MDPI, Basel, Switzerland. This article is an open access article distributed under the terms and conditions of the Creative Commons by Attribution (CC-BY) license (http://creativecommons.org/licenses/by/4.0/).
